# Síndrome Coronariana Aguda no Brasil: Registro dos Fatores Predisponentes e Perfil Populacional em um Instituto Cardiológico Público de Referência Nacional

**DOI:** 10.36660/abc.20240165

**Published:** 2024-11-28

**Authors:** Kaliana Nascimento, Hugo Ribeiro Ramadan, Bruno Mendonça Baccaro, Vinicius Vaz de Sales Bicalho, Italo Menezes Ferreira, Louis Nakayama Ohe, Vitor Sobreira Souza Santos, Fausto Feres, Kleber Franchini, Ari Timerman, Diandro Marinho Mota

**Affiliations:** 1 Instituto Dante Pazzanese de Cardiologia São Paulo SP Brasil Instituto Dante Pazzanese de Cardiologia, São Paulo, SP – Brasil

**Keywords:** Doenças Cardiovasculares, Síndrome Coronariana Aguda, Isquemia Miocárdica, Doença das Coronárias

## Abstract

**Fundamentos:**

A síndrome coronariana aguda (SCA) é uma das principais causas de mortalidade no mundo. Conhecer os fatores predisponentes é essencial para a prevenção desta.

**Objetivos:**

Descrever as características etiológicas e epidemiológicas da população com SCA admitida em um pronto-socorro do Estado de São Paulo.

**Métodos:**

A coorte prospectiva realizada com base nos registros de prontuário eletrônico de um instituto cardiológico público localizado no Estado de São Paulo descreve 5.580 pacientes internados com SCA entre agosto de 2018 e outubro de 2022. Avaliou-se as principais características epidemiológicas, a associação entre confirmação de SCA e escores de risco, eventos adversos durante o internamento e no seguimento de 30 dias após a alta hospitalar. O nível de significância foi de 5%.

**Resultados:**

Os principais fatores associados a SCA foram hipertensão (80,38%), obesidade ou sobrepeso (72,47%) e doença arterial coronariana prévia (59,11%). No escore Grace, 65,10% foram considerados de baixo risco, enquanto 81,34% no TIMI e 71,16% no HEART foram identificados como risco moderado. O cateterismo representou 84,93% dos métodos diagnósticos. O tratamento clínico foi a estratégia adotada em 46,47% dos casos. Na evolução em 30 dias, 3,10% apresentaram sangramento maior, 7,86% infarto/reinfarto, 5,55% acidente vascular encefálico e 2,53% evoluíram para óbito.

**Conclusão:**

Os resultados do maior registro de SCA brasileiro até o momento evidenciam o impacto dos fatores de risco potencialmente modificáveis na ocorrência de eventos isquêmicos na população local. Os achados podem contribuir para o desenvolvimento de políticas públicas visando a prevenção e controle da carga da doença isquêmica no país.

## Graphical abstract

**Figura Central:**
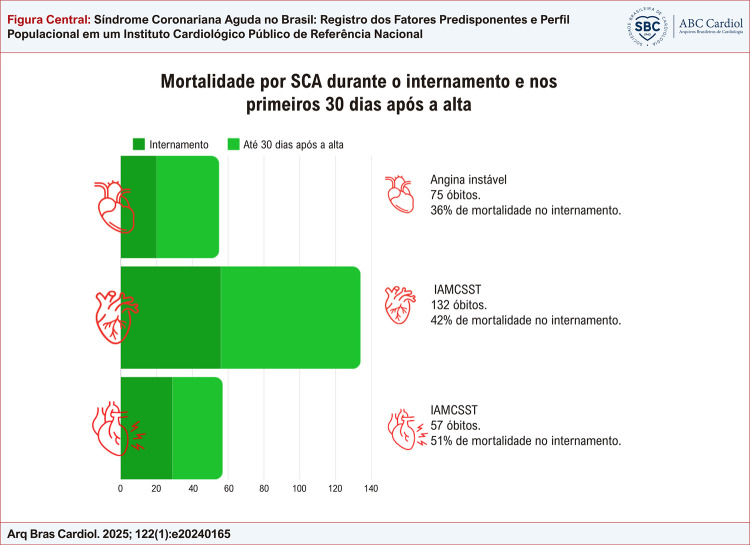
: Síndrome Coronariana Aguda no Brasil: Registro dos Fatores Predisponentes e Perfil Populacional em um Instituto Cardiológico Público de Referência Nacional

SCA: síndrome coronariana aguda; IAMCSST: infarto agudo do miocárdio com supradesnivelamento do segmento ST.

## Introdução

A doença cardiovascular (DCV) ocupa o primeiro lugar em mortalidade no mundo. Dados da Organização Mundial de Saúde (OMS) de 2019 revelam que aproximadamente 18 milhões de óbitos foram por DCV. Dentre as principais causas de morte por DCV, a doença cardíaca isquêmica (DCI) representa a mais importante. Em 2019 foram registrados em torno de 9 milhões de óbitos por DCI,^1^ sendo mais de 100.000 no Brasil, representando 43% de todas as mortes por DCV.^2,3^ No registro nacional DATASUS 2021, foram descritos aproximadamente 115.000 óbitos por DCI, refletindo o cenário global. Dentre as principais causas para os números alarmantes estão fatores de risco preveníveis como o sedentarismo, o tabagismo, a obesidade e o mau controle da hipertensão e do diabetes. O estudo INTERHEART,^4^ evidencia os fatores citados como principais barreiras a serem ultrapassadas, e apesar dos progressos tecnológicos e dos esforços das instituições de saúde, o controle de tais fatores segue desafiador.

No Brasil, o Sudeste detém a maior porcentagem de óbitos por DCI (47%), com a maior representatividade pelo Estado de São Paulo (57%).^5^

É fundamental compreender o perfil epidemiológico e demográfico dos pacientes internados por síndrome coronariana aguda (SCA) para entender os fatores modificáveis da DCI.

O último grande registro brasileiro de SCA data de 2013 e contava com 2693 pacientes,^6^ quando ainda não se utilizava a troponina ultrassensível para o diagnóstico de infarto agudo do miocárdio (IAM). O presente estudo engloba a incidência de angina instável (AI), infarto agudo do miocárdio sem supradesnivelamento do segmento ST (IAMSSST) e infarto agudo do miocárdio com supradesnivelamento do segmento ST (IAMCSST) em pacientes internados em um instituto cardiológico de referência nacional, localizado no Estado de São Paulo, e com média de 3000 atendimentos cardiológicos por mês. O objetivo é apresentar o perfil de classificação da SCA através da instituição avaliada, bem como de comorbidades e das principais estratégias terapêuticas e de tratamento utilizadas ao momento de realização do estudo e os principais desfechos ao longo dos 30 dias de seguimento. Almeja-se contribuir com a comunidade científica no entendimento de como prevenir a ocorrência de SCA, tema ainda desafiador no século XXI.

## Métodos

### Design do estudo e seleção de pacientes

Este estudo consiste em uma coorte prospectiva realizada num instituto cardiológico localizado no Estado de São Paulo. Os pacientes com diagnóstico de SCA eram inseridos no registro, no momento da admissão hospitalar e acompanhados com ligações telefônicas ou preenchimento de formulário enviado por mensagem eletrônica para seguimento de 30 dias. O período de inclusão abrange agosto de 2018 a outubro de 2022. O estudo foi aprovado pelo comitê de ética da instituição. No total, foram avaliados 5580 pacientes hospitalizados, todos com suspeita ou diagnóstico confirmado de SCA, incluindo AI, IAMSSST e IAMCSST. A metodologia diagnóstica da SCA seguiu as diretrizes brasileiras de SCA e IAM vigentes no momento da coorte.^7-9^

O diagnóstico específico de IAM é baseado nas diretrizes da quarta definição de infarto, que considera a elevação ou queda dos níveis de troponina de alta sensibilidade acima do percentil 99. Este critério deve estar associado a pelo menos um indicativo de isquemia, que pode ser: sintomas de isquemia miocárdica, alterações eletrocardiográficas indicativas de isquemia, a presença de nova onda Q no ECG, resultados de coronariografia que mostrem trombose ou evidências de perda de parede ou viabilidade muscular em exames de imagem não invasivos.^10^

A AI foi definida como isquemia miocárdica em repouso ou com mínimo esforço na ausência de injúria aguda (curva de troponina). Caracteriza-se por achados clínicos específicos de angina prolongada (mais de 20 minutos) em repouso, angina de início recente e intensa, angina aumentando em frequência e duração, ou menor em limiar de tolerabilidade; ou angina após um episódio recente de infarto do miocárdio.^11^

Para a análise, foi utilizada a troponina I de alta sensibilidade do kit VITROS (Ortho Clinical Diagnostics), com um limiar de quantificação estabelecido em 1,5 ng/L e um limiar para o 99º percentil fixado em 11 ng/L. Os intervalos de amostragem adotados foram de 0 a 1 hora. É importante ressaltar que até outubro de 2021, a instituição utilizava um tipo de troponina que não possuía a classificação de alta sensibilidade (Troponina Biomerieux).

### Dados clínicos e desfechos

Foram avaliadas as principais características epidemiológicas da população supracitada, dentre elas a idade, gênero, sobrepeso, obesidade, dislipidemia, diabetes mellitus insulinodependente ou não, tabagismo, doença renal crônica e histórico de DCI.

Os pacientes foram classificados nos escores de dor torácica/SCA (HEART score, TIMI risk e GRACE). O objetivo foi avaliar a incidência de SCA de acordo com os principais métodos de investigação (cateterismo, cintilografia miocárdica, angiotomografia de coronárias, teste ergométrico, ressonância miocárdica ou ecocardiograma com estresse) e associar com os fatores epidemiológicos e escores já descritos. Apresentamos também as principais estratégias de tratamento (clínica, angioplastia e cirurgia) e a taxa de sangramento maior, acidente vascular encefálico, infarto e morte durante o período de internamento até 30 dias após a alta hospitalar.

### Análise estatística

As variáveis contínuas foram descritas através de mediana e intervalo interquartil e as variáveis categóricas foram apresentadas através de frequências absolutas e relativas. A associação entre os grupos, para as variáveis categóricas, foi avaliada através do teste Qui-Quadrado. A comparação entre os grupos, nas variáveis contínuas, foi realizada através do teste de Kruskal-Wallis, seguido do teste de Dunn, já que o teste de normalidade de Kolmogorov-Smirnov mostrou que essas variáveis não têm distribuição normal. O nível de significância adotado foi de 5%. Utilizou-se o software *R Core Team (2023)*.

## Resultados

A Tabela 1 apresenta o perfil clínico e demográfico do total de 5.580 pacientes e também por grupo (AI, IAMSSST e IAMCSST), além da comparação entre os três grupos, com mediana de idade de 63 anos. Os pacientes grupo IAM sem ST são mais velhos que os pacientes dos outros dois grupos (p-valor<0,001).

**Tabela 1:** – Perfil demográfico e clínico

Variável	Angina Instável	IAMSSST	IAMCSST	SCA	p-valor
N (%)	3124 (55,99)	1944 (34,84)	512 (9,18)	5580 (100)	
Idade (anos), mediana (q1-q3)	63 (56-70)	63 (55-69)	65 (58-72)	62 (55-70)	<0.001
Sexo feminino, n (%)	1153 (36,90)	692 (35,59)	160 (31,25)	2005 (35,93)	0,044
IMC médio (Kg/m^2^), mediana (q1-q3)	27,6 (24,8-30,7)	27,7 (25-30,8)	27,4 (24,6-0,4	27,3 (24,5-30,6)	<0.001
Diabetes, n (%)	1836 (58,77)	1075 (55,29)	245 (47,85)	3156 (56,55)	
DMNID, n	1344 (73,20)	727 (67,62)	186 (75,91)	2257 (71,51)	<0,001
DMID, n	492 (26,90)	348 (32,37)	59 (24,08)	899 (28,48)	
Hipertensão, n (%)	2551 (81,66)	1572 (80,86)	362 (70,70)	4485 (80,38)	<0,001
Sobrepeso/obesidade, n (%)	2312 (74,00)	1372 (70,57)	360 (70,31)	4044 (72,47)	
Sobrepeso, n (%)	1371 (59,29)	840 (61,22)	217 (60,27)	2428 (60,03)	0,062
Obesidade GI, n (%)	676 (29,23)	385 (28,06)	100 (27,77)	1161 (28,70)	
Obesidade GII, n (%)	179 (7,74)	100 (7,28)	36 (10,00)	315 (7,78)	
Obesidade GIII, n (%)	86 (3,71)	47 (3,42)	7 (1,94)	140 (3,46)	
Tabagismo (ou cessou recentemente), n (%)	587 (18,79)	394 (20,26)	168 (32,81)	1149 (20,59)	<0,001
Dislipidemia, n (%)	1717 (54,96)	1065 (54,78)	203 (39,64)	2985 (53,49)	<0,001
Doença renal crônica					
Estágio I, n (%)	1362 (43,59)	673 (34,61)	228 (44,52)	2263 (40,55)	<0,001
Estágio II, n (%)	1106 (35,39)	660 (33,94)	160 (31,24)	1926 (34,51)	
Estágio III, n (%)	344 (11,00)	301 (15,47)	51 (9,95)	696 (12,46)	
Estágio IV, n (%)	203 (6,49)	194 (9,97)	40 (7,80)	437 (7,82)	
Estágio V ou TRS, n (%)	109 (3,48)	116 (5,96)	33 (6,44)	258 (4,61)	
DAC prévia, n (%)	1932 (61,83)	1162 (59,76)	205 (40,03)	3299 (59,11)	<0,001

IAMSSST: infarto agudo do miocárdio sem supradesnivelamento do segmento ST; IAMCSST: infarto agudo do miocárdio com supradesnivelamento do segmento ST; SCA: síndrome coronariana aguda; IMC: índice de massa corporal; DMNID:diabetes mellitus não insulinodependente; DMID: diabetes mellitus insulinodependente; DAC: doença arterial coronariana.

A prevalência do sexo feminino (36%) é menor no grupo IAMSSST comparado aos outros dois grupos (p-valor <0,001). Apesar de pouca diferença entre os grupos em relação ao índice de massa corporal (IMC) os testes de Kruskal-Wallis e Dunn mostraram que o grupo AI apresenta maior IMC que os outros dois grupos (p-valor<;0,001). Dentre os diabéticos (57%), há maior associação do diabetes mellitus não insulinodependente com AI e IAMCSST, enquanto o diabetes mellitus insulinodependente está mais associado com IAMSSST.

A prevalência de hipertensão (HAS) (80%) é menor no grupo IAMCSST comparado aos outros dois grupos (p-valor <0,001). Não foram observadas associações os grupos em relação ao sobrepeso/obesidade (p-valor = 0,062). Os tabagistas ou que cessaram recentemente perfazem 21% dos pacientes, com prevalência maior no grupo IAMCSST comparado aos outros dois grupos (p-valor <0,001).

A prevalência de dislipidemia é menor no grupo IAMCSST comparado aos outros dois grupos (p-valor <0,001). A prevalência de doença arterial coronariana (DAC) prévia (59%) é menor no grupo infarto com supra comparado aos outros dois grupos (p-valor <0,001).

A Tabela 2 apresenta a classificação de risco, estratégia de investigação e tratamento para o total de pacientes e por grupo, além da comparação entre os grupos.

**Tabela 2:** – Classificação de risco, estratégia de investigação e tratamento

Variável	Angina Instável	IAMSSST	IAMCSST	SCA	p-valor
**Classificação de risco**					
GRACE baixo risco, n (%)	2432 (77,84)	987 (50,76)	214 (41,79)	3633 (65,10)	<0,001
GRACE risco moderado, n (%)	627 (20,06)	727 (37,39)	198 (38,66)	1552 (27,81)	
GRACE alto risco, n(%)	65 (2,07)	230 (11,82)	100 (19,52)	395 (7,08)	
TIMI baixo risco, n (%)	415 (13,27)	148 (7,60)	49 (9,56)	612 (10,97)	<0,001
TIMI risco moderado, n (%)	2631 (84,21)	1517 (78,02)	391 (76,36)	4539 (81,34)	
TIMI alto risco, n (%)	78 (2,49)	279 (14,34)	72 (14,05)	429 (7,69)	
HEART baixo risco, n (%)	368 (11,77)	60 (3,08)	-	428 (7,67)	<0,001
HEART risco moderado, n (%)	2616 (83,73)	1355 (69,69)	-	3971 (71,16)	
HEART alto risco (%)	148 (4,73)	529 (27,20)	-	677 (12,13)	
**Estratégia de investigação**					
Cateterismo cardíaco (CATE), n (%)	2466 (78,93)	1783 (91,71)	490 (95,69)	4739 (84,93)	<0,001
Cintilografia miocárdica, n (%)	392 (12,54)	59 (3,02)	2 (0,38)	453 (8,12)	<0,001
Angiotomografia de coronárias, n (%)	112 (3,58)	10 (0,50)	-	122 (2,19)	NC
Ressonância magnética (REMA), n (%)	18 (0,57)	9 (0,45)	2 (0,38)	29 (0,52)	NC
Teste ergométrico, n (%)	9 (0,28)	-	-	9 (0,16)	NC
Ecocardiograma com estresse, n (%)	-	-	-	-	NC
**Tratamento realizado**					
Clínico, n (%)	1692 (54,15)	792 (40,73)	109 (21,28)	2593 (46,47)	<0,001
Angioplastia, n (%)	1068 (34,18)	912 (46,90)	377 (73,62)	2357 (42,24)	<0,001
Cirurgia de revascularização miocárdica, n (%)	364 (11,64)	240 (12,34)	26 (5,07)	630 (11,29)	<0,001

NC: não calculado devido ao baixo número de pacientes; IAMSSST: infarto agudo do miocárdio sem supradesnivelamento do segmento ST; IAMCSST: infarto agudo do miocárdio com supradesnivelamento do segmento ST; SCA: síndrome coronariana aguda.

Quanto à evolução para o total de pacientes e por grupo (Tabela 3), temos os seguintes achados: 3% dos pacientes apresentaram sangramento maior, sendo um pouco menos prevalente no grupo da AI, mesmo padrão seguido pelo acidente vascular encefálico (p-valor 0,037 e 0,007, respectivamente).

**Tabela 3:** – Evolução

Variável	Angina Instável	IAMSSST	IAMCSST	SCA	p-valor
Evolução durante internação hospitalar					
Sangramento maior (TIMI), n (%)	80 (2,56)	74 (3,80)	18 (3,51)	172 (3,08)	0,037
Acidente vascular encefálico, n (%)	13 (0,41)	22 (1,13)	6 (1,17)	41 (0,73)	0,007
Infarto/reinfarto, n(%)	50 (1,60)	37 (1,90)	15 (2,92)	102 (1,83)	0,109
Morte, n (%)	20 (0,64)	56 (2,88)	29 (5,66)	105 (1,88)	<0,001
**Evolução em até 30 dias, após alta**					
Sangramento maior (TIMI), n (%)	118 (3,77)	45 (2,31)	10 (1,95)	173 (3,10)	0,004
Acidente vascular encefálico, n (%)	195 (6,24)	87 (4,47)	19 (3,71)	310 (5,55)	0,005
Infarto/reinfarto, n(%)	254 (8,13)	160 (8,23)	24 (4,68)	439 (7,86)	0,020
Morte, n (%)	35 (1,12)	78 (4,01)	28 (5,47)	141 (2,53)	<0,001
Tempo médio de internação hospitalar, dias	6,42	7,55	7,55	7,17	

IAMSSST: infarto agudo do miocárdio sem supradesnivelamento do segmento ST; IAMCSST: infarto agudo do miocárdio com supradesnivelamento do segmento ST; SCA: síndrome coronariana aguda.

Infarto/reinfarto foi presente em 1,8% dos pacientes sendo mais prevalente com grupo IAMCSST (p-valor = 0,019). 1,9% dos pacientes foram a óbito, sendo mais presente no grupo IAMCSST seguido do IAMSSST (p-valor<0,001) (Figura 1).

Após 30 dias de alta, 3% dos pacientes apresentaram sangramento maior, sendo um pouco menos prevalente no grupo de AI (p-valor 0,004). O acidente vascular encefálico foi presente em 5% dos pacientes, sendo um pouco menos prevalente no grupo de AI (p-valor = 0,005);

Reinfarto ocorreu em 8% dos pacientes sendo menos prevalente com grupo IAMCSST (p-valor = 0,020). 2,5% dos pacientes foram a óbito, sendo mais presente nos grupos com IAM (p-valor<0,001). A figura central resume os principais achados descritos acima.

## Discussão

O presente registro é o maior já divulgado sobre SCA até a data de publicação. De forma detalhada e consistente é possível perceber a influência dos fatores de risco predisponentes na incidência de SCA.

As principais comorbidades encontradas (hipertensão, obesidade ou sobrepeso, diabetes e dislipidemia) estão presentes em pelo menos metade da população e seguem a tendência mundial, existindo inclusive diretrizes globalmente validadas para a prevenção de tais fatores de risco.^12^ No tangente à obesidade, observa-se a prevalência em 70% dos avaliados, muito acima dos 39% descritos pela OMS em 2016, o que eleva o patamar de risco cardiovascular (RCV) da nossa população.^13^ Em 2019, um registro mundial já alertava para a obesidade como contribuinte para cinco milhões de mortes globalmente.^14^De forma não surpreendente, hipertensão, DAC, insuficiência cardíaca aumentam com a obesidade.

A presença de mais de 50% indivíduos diabéticos é também alarmante. Em 2017, o estudo *Pure* alertou sobre a correlação alimentar, principalmente rica em carboidratos com doença e morte cardiovascular.^15^ Dados na literatura reforçam que o aumento do RCV já está presente no pré-diabético, e que a DCV é a principal causa de morte no indivíduo diabético.^16^ Em uma coorte realizada nos Estados Unidos, a diabetes representa um aumento de 18% de morte cardiovascular.^17^ Nosso estudo reforça a necessidade de estratégias para prevenção primária e secundária da DM, visto a prevalência dessa doença nos portadores de DAC.

Setenta por cento dos pacientes representados no estudo são hipertensos. Uma metanálise revelou a associação da HAS com morte por DAC em todas as idades, principalmente quando associada ao tempo de doença e a elevação em 20mmHg na pressão sistólica e 10mmHg na pressão diastólica.^18^ Dados de 2019 evidenciam que a HAS foi a causa principal de 1,16 milhões de óbitos, além de 21,5 milhões de perda de expectativa de vida ajustada pela idade.^19^ Sob a ótica de tais dados, o presente registro traz um alerta quanto à necessidade de medidas para prevenir a HAS, dar o diagnóstico precoce e evitar a inércia terapêutica.

Aproximadamente metade da população estudada (40%) havia apresentado ao menos um episódio de SCA. Uma coorte estadunidense avaliou aproximadamente 240 mil pacientes com primeiro evento de SCA e o risco de reincidência, e revelou que 40% da população apresenta risco de novo evento ainda no primeiro ano da alta hospitalar.^20^ Um registro brasileiro com quinze mil pacientes avaliou os principais fatores associados à reincidência da SCA, destacando a adesão medicamentosa como um dos principais fatores contribuintes para reincidência de SCA. Para exemplificar, 16% dos avaliados não tomava nenhuma das medicações recomendadas e 11,8% usavam apenas 1 das medicações em apenas 4 anos do evento.^21^ Portanto, podemos colocar a má adesão medicamentosa como um novo fator de risco a considerarmos nos pacientes.

Todos os fatores anteriormente mencionados podem ser prevenidos por meio do aprimoramento de políticas públicas voltadas à prevenção primária das DCs. Essas políticas englobam desde campanhas educativas sobre a relevância de manter uma rotina de exercícios físicos e uma dieta equilibrada. A OMS recomenda a prática de 150 a 300 minutos de atividade de intensidade moderada por semana ou 75 a 150 minutos de atividade intensa por semana.^22^

Outro ponto fundamental é a adesão correta à medicação, a cessação do hábito de fumar, e a implementação de um atendimento personalizado. Todas essas medidas devem ser iniciadas nas Unidades de Saúde da Família, as quais desempenham um papel crucial na detecção precoce dos principais fatores de risco para doenças na população assistida, além de estabelecerem um acompanhamento na mudança do estilo de vida dos indivíduos. Vale destacar que a utilização isolada dos escores de risco (GRACE, TIMI e HEART) isoladamente não apresentaram boa acurácia para a distinção entre AI e Infarto. Estudos já sugerem classificações combinadas para aumentar a acurácia também do diagnóstico angiográfico, inclusive com os outros escores apresentados no registro.^23^

O desempenho dos escores de risco tem sido questionado. Evidências recentes têm demonstrado que o julgamento clínico associado a troponina de alta sensibilidade e eletrocardiograma têm maior sensibilidade e VPN que o escore de TIMI.^24^

Nossa prática clínica evidencia uma alta frequência no uso de cateterismo cardíaco, refletindo diretamente a realidade epidemiológica da população atendida. Observamos uma prevalência significativamente elevada de DAC pré-existente entre os pacientes, aproximando-se de 60%. Este alto índice de DAC prévia na população justifica a frequente recorrência ao cateterismo como ferramenta diagnóstica e terapêutica primária em nossa prática médica.

No seguimento de 30 dias ressalta-se a presença de sangramento maior, acidente vascular cerebral isquêmico, infarto e óbito associado à AI. Sabe-se que atualmente o diagnóstico de AI é questionado na literatura,^25^ o que amplia os parâmetros de identificação, mas ao mesmo tempo torna mais difícil o diagnóstico preciso e o manejo terapêutico imediato, estando o paciente mais propenso a eventos tromboembólicos.

### Limitações do estudo

A coleta de dados iniciou em 2019, momento em que a instituição ainda não utilizava o kit de troponina de alta sensibilidade, somente este sendo padronizado em 2021. Tal fator pode ser subestimado o diagnóstico de infarto, bem como superestimado o diagnóstico de AI. Para a composição de grande parte do registro as informações eram colhidas diretamente com os pacientes, tendo como limitante a subjetividade da compreensão do questionamento, bem como um mascaramento da condição patológica dos pacientes, pois muitos pacientes que eram admitidos na instituição apresentavam um frágil histórico de acompanhamento médico, desconhecendo até mesmo ser portadores de comorbidades identificadas na atenção básica, como hipertensão e diabetes. Desse modo, estima-se que a prevalência das comorbidades supracitadas seja ainda maior do que a descrita no presente estudo. Acrescenta-se ainda a dificuldade de classificação dos entrevistados quanto à prática de atividade física, pois as respostas divergiram na medida em que eram novamente questionados durante o seguimento. Pela possibilidade de não representação fidedigna dos dados, os autores optaram por não divulgar tal informação, mas ressaltando a importância do combate ao sedentarismo como medida de prevenção ao risco cardiovascular. Não foi possível classificar inicial a origem de todos os pacientes admitidos no pronto-socorro do serviço e o tempo total dos sintomas desde o primeiro atendimento em caso de encaminhamento. Tal fato se deve indisponibilidade dos registros do SUS com a avaliação completa do paciente até este dar entrada no pronto-socorro da instituição. Por fim, ressalta-se que o tempo de seguimento possa ter sido insuficiente para concluir sobre os principais desfechos cardiovasculares, embora os resultados encontrados sejam endossados pelos principais estudos em síndrome coronariana na comunidade científica.

## Conclusão

O registro acima contribui para elucidar quais as estratégias a serem utilizadas na prevenção primária da SCA, auxiliando na geração de políticas públicas para tais. Destaca-se ainda a necessidade de novos estudos que incluam as demais regiões do país, a fim de traduzir com maior precisão a realidade de saúde nacional.
