# Supplementary material for: Síndrome Coronariana Aguda no Brasil: Registro dos Fatores Predisponentes e Perfil Populacional em um Instituto Cardiológico Público de Referência Nacional
Source: Arq Bras Cardiol. 2024 Nov 28;122(1):e20240165. [Article in Portuguese] doi: 10.36660/abc.20240165 (PMC11661571; doi:10.36660/abc.20240165)
Supplement: Translation

# Translation: Acute Coronary Syndrome in Brazil: Registration of Predisposing Factors and Population Profile in a National Public Reference Cardiological Institute

**DOI:** 10.36660/abc.20240165i

**Authors:** Kaliana Nascimento, Hugo Ribeiro Ramadan, Bruno Mendonça Baccaro, Vinicius Vaz de Sales Bicalho, Italo Menezes Ferreira, Louis Nakayama Ohe, Vitor Sobreira Souza Santos, Fausto Feres, Kleber Franchini, Ari Timerman, Diandro Marinho Mota

**Affiliations:** 1 Brazil Instituto Dante Pazzanese de Cardiologia, São Paulo, SP – Brazil

**Keywords:** Cardiovascular Diseases, Acute Coronary Syndrome, Myocardial Ischemia, Coronary Disease

## Abstract

**Background:**

Acute coronary syndrome (ACS) is one of the leading causes of mortality worldwide. Knowing the predisposing factors is essential for preventing it.

**Objectives:**

To describe the etiological and epidemiological characteristics of the population with ACS admitted to an emergency room in the State of São Paulo.

**Methods:**

The prospective cohort study, based on electronic medical records from a public cardiology institute located in the state of São Paulo, Brazil, describes 5,580 patients hospitalized with ACS between August 2018 and October 2022. The main epidemiological characteristics, the association between confirmed ACS and risk scores, and adverse events during hospitalization and in the 30-day follow-up after hospital discharge were evaluated. The significance level was set at 5%.

**Results:**

The main factors associated with ACS were hypertension (80.38%), obesity or overweight (72.47%), and previous coronary artery disease (CAD) (59.11%). In the GRACE score, 65.10% were considered low risk, while 81.34% in the TIMI and 71.16% in the HEART were identified as moderate risk. Catheterization represented 84.93% of the diagnostic methods. Clinical treatment was the strategy adopted in 46.47% of the cases. In the 30-day evolution, 3.10% presented major bleeding, 7.86% infarction/reinfarction, 5.55% stroke, and 2.53% evolved to death.

**Conclusion:**

The results of the largest Brazilian ACS registry to date highlight the impact of potentially modifiable risk factors on the occurrence of ischemic events in the local population. The findings may contribute to the development of public policies aimed at preventing and controlling the burden of ischemic disease in the country.

## Introduction

Cardiovascular disease (CVD) is the leading cause of death worldwide. Data from the World Health Organization (WHO) from 2019 reveals that approximately 18 million deaths were due to CVD. Among the main causes of death from CVD,^1^ ischemic heart disease (IHD) is the most important. In 2019, around 9 million deaths from IHD were recorded (The top 10 causes of death.” World Health Organization (WHO), December 9, 2020.), with more than 100,000 in Brazil, representing 43% of all deaths from CVD.^2,3^ In the national registry DATASUS 2021, approximately 115,000 deaths from IHD were described, reflecting the global scenario. Among the main causes for the alarming numbers are preventable risk factors such as a sedentary lifestyle, smoking, obesity, and poor control of hypertension and diabetes. The INTERHEART study,^4^ highlights the factors cited as the main barriers to be overcome, and despite technological advances and the efforts of health institutions, controlling such factors remains challenging.

In Brazil, the Southeast has the highest percentage of deaths due to IHD (47%), with the largest representation in the State of São Paulo (57%).^5^

It is essential to understand the epidemiological and demographic profile of patients hospitalized for acute coronary syndrome (ACS) to understand the modifiable factors of IHD.

The last large Brazilian registry of ACS dates back to 2013 and included 2,693 patients,^6^ when high-sensitivity troponin was not yet used for the diagnosis of acute myocardial infarction (AMI). The present study encompasses the incidence of unstable angina (UA), non-ST-segment elevation myocardial infarction (NSTEMI), and ST-segment elevation myocardial infarction (STEMI) in patients admitted to a nationally renowned cardiology institute located in the state of São Paulo, with an average of 3,000 cardiology consultations per month. The objective is to present the ACS classification profile according to the institution evaluated, as well as comorbidities, the main therapeutic and treatment strategies used at the time of the study, and the main outcomes over the 30 days of follow-up. The aim is to contribute to the scientific community in understanding how to prevent the occurrence of ACS, a topic that is still challenging in the 21st century.

## Methods

### Study design and patient selection

This study consists of a prospective cohort study conducted at a cardiology institute located in the state of São Paulo. Patients diagnosed with ACS were included in the registry at the time of hospital admission and followed up by telephone calls or by completing a form sent by email for a 30-day follow-up. The inclusion period covers August 2018 to October 2022. The institution’s ethics committee approved the study. In total, 5580 hospitalized patients were evaluated, all with suspected or confirmed diagnoses of ACS, including UA, NSTEMI, and STEMI. The diagnostic methodology for ACS followed the Brazilian guidelines for ACS and AMI in force at the time of the cohort study.^7-9^

The specific diagnosis of AMI is based on the guidelines of the fourth definition of infarction, which considers the elevation or fall of high-sensitivity troponin levels above the 99th percentile. This criterion must be associated with at least one indication of ischemia, which may be symptoms of myocardial ischemia, electrocardiographic changes indicative of ischemia, the presence of a new Q wave on the ECG, coronary angiography results showing thrombosis or evidence of loss of wall or muscle viability in non-invasive imaging tests.^10^

UA was defined as myocardial ischemia at rest or with minimal exertion in the absence of acute injury (troponin curve). It is characterized by specific clinical findings of prolonged angina (more than 20 minutes) at rest, recent onset and severe angina, angina increasing in frequency and duration or lower in tolerability threshold, or angina after a recent episode of myocardial infarction.^11^

For the analysis, high-sensitivity troponin I from the VITROS kit (Ortho Clinical Diagnostics) was used, with a quantification threshold set at 1.5 ng/L and a threshold for the 99th percentile set at 11 ng/L. The sampling intervals adopted were from 0 to 1 hour. It is important to note that until October 2021, the institution used a type of troponin that did not have the high-sensitivity classification (Troponin Biomerieux).

### Clinical data and outcomes

The main epidemiological characteristics of the population mentioned above were evaluated, including age, gender, overweight, obesity, dyslipidemia, insulin-dependent or non-insulin-dependent diabetes mellitus, smoking, chronic kidney disease, and history of IHD.

Patients were classified according to chest pain/ACS scores (HEART score, TIMI risk, and GRACE). The objective was to evaluate the incidence of ACS according to the main investigation methods (catheterization, myocardial scintigraphy, coronary angiotomography, exercise stress test, myocardial resonance imaging, or stress echocardiography) and to associate it with the epidemiological factors and scores previously described. We also present the main treatment strategies (clinical, angioplasty, and surgery) and the rate of major bleeding, stroke, infarction, and death during the hospital stay up to 30 days after hospital discharge.

### Statistical analysis

Continuous variables were described using median and interquartile range, and categorical variables were presented using absolute and relative frequencies. The association between groups for categorical variables was assessed using the Chi-Square test. The comparison between groups for continuous variables was performed using the Kruskal-Wallis test, followed by the Dunn test, since the Kolmogorov-Smirnov normality test showed that these variables do not have a normal distribution. The significance level adopted was 5%. The R Core Team (2023) software was used.

## Results

Table 1 presents the clinical and demographic profile of a total of 5,580 patients and also by group (UA, NSTEMI, and STEMI), in addition to the comparison between the three groups, with a median age of 63 years. Patients in the non-ST AMI group are older than patients in the other two groups (p-value <0.001).

**Table 1:** – Demographic and clinical profile

Variable	Unstable Angina	NSTEMI	STEMI	ACS	p-value
N (%)	3124 (55.99)	1944 (34.84)	512 (9.18)	5580 (100)	
Age (years), median (q1-q3)	63 (56-70)	63 (55-69)	65 (58-72)	62 (55-70)	<0.001
Female, n (%)	1153 (36.90)	692 (35.59)	160 (31.25)	2005 (35.93)	0.044
Average BMI (Kg/m^2^), median (q1-q3)	27.6 (24.8-30.7)	27.7 (25-30.8)	27.4 (24.6-0.4	27.3 (24.5-30.6)	<0.001
Diabetes, n (%)	1836 (58.77)	1075 (55.29)	245 (47.85)	3156 (56.55)	
NIDDM, n	1344 (73.20)	727 (67.62)	186 (75.91)	2257 (71.51)	<0.001
IDDM, n	492 (26.90)	348 (32.37)	59 (24.08)	899 (28.48)	
Hypertension, n (%)	2551 (81.66)	1572 (80.86)	362 (70.70)	4485 (80.38)	<0.001
Overweight/obesity, n (%)	2312 (74.00)	1372 (70.57)	360 (70.31)	4044 (72.47)	
Overweight, n (%)	1371 (59.29)	840 (61.22)	217 (60.27)	2428 (60.03)	0.062
Obesity GI, n (%)	676 (29.23)	385 (28.06)	100 (27.77)	1161 (28.70)	
Obesity GII, n (%)	179 (7.74)	100 (7.28)	36 (10.00)	315 (7.78)	
Obesity GIII, n (%)	86 (3.71)	47 (3.42)	7 (1.94)	140 (3.46)	
Smoking (or recently quit), n (%)	587 (18.79)	394 (20.26)	168 (32.81)	1149 (20.59)	<0.001
Dyslipidemia, n (%)	1717 (54.96)	1065 (54.78)	203 (39.64)	2985 (53.49)	<0.001
Chronic kidney disease					
Stage I, n (%)	1362 (43.59)	673 (34.61)	228 (44.52)	2263 (40.55)	<0.001
Stage II, n (%)	1106 (35.39)	660 (33.94)	160 (31.24)	1926 (34.51)	
Stage III, n (%)	344 (11.00)	301 (15.47)	51 (9.95)	696 (12.46)	
Stage IV, n (%)	203 (6.49)	194 (9.97)	40 (7.80)	437 (7.82)	
Stage V or TRS, n (%)	109 (3.48)	116 (5.96)	33 (6.44)	258 (4.61)	
Previous CAD, n (%)	1932 (61.83)	1162 (59.76)	205 (40.03)	3299 (59.11)	<0.001

ACS: Acute coronary syndrome; BMI: body mass index; NIDDM: non-insulin dependent diabetes mellitus; IDDM: insulin-dependent diabetes mellitus; CAD: coronary artery disease; NSTEMI: non-ST-segment elevation myocardial infarction; STEMI: ST-segment elevation myocardial infarction.

The prevalence of females (36%) is lower in the NSTEMI group compared to the other two groups (p-value <0.001). Despite little difference between the groups in relation to body mass index (BMI), the Kruskal-Wallis and Dunn tests showed that the UA group has a higher BMI than the other two groups (p-value <0.001). Among people with diabetes (57%), there is a greater association of non-insulin dependent diabetes mellitus with UA and STEMI, while insulin-dependent diabetes mellitus is more associated with NSTEMI.

The prevalence of hypertension (80%) was lower in the STEMI group compared to the other two groups (p-value <0.001). No associations were observed between the groups in relation to overweight/obesity (p-value = 0.062). Smokers or those who recently quit smoking accounted for 21% of patients, with a higher prevalence in the STEMI group compared to the other two groups (p-value <0.001).

The prevalence of dyslipidemia is lower in the STEMI group compared to the other two groups (p-value <0.001). The prevalence of previous CAD (59%) is lower in the infarction with the supra group compared to the other two groups (p-value <0.001).

Table 2 presents the risk classification, investigation and treatment strategy for all patients and by group, in addition to the comparison between the groups.

**Table 2:** – Risk classification, investigation, and treatment strategy

Variable	Unstable Angina	NSTEMI	STEMI	ACS	p-value
**Risk classification**					
GRACE low risk, n (%)	2432 (77.84)	987 (50.76)	214 (41.79)	3633 (65.10)	<0.001
GRACE moderate risk, n (%)	627 (20.06)	727 (37.39)	198 (38.66)	1552 (27.81)	
GRACE high risk, n (%)	65 (2.07)	230 (11.82)	100 (19.52)	395 (7.08)	
TIMI low risk, n (%)	415 (13.27)	148 (7.60)	49 (9.56)	612 (10.97)	<0.001
TIMI moderate risk, n (%)	2631 (84.21)	1517 (78.02)	391 (76.36)	4539 (81.34)	
TIMI high risk, n (%)	78 (2.49)	279 (14.34)	72 (14.05)	429 (7.69)	
HEART low risk, n (%)	368 (11.77)	60 (3.08)	-	428 (7.67)	<0.001
HEART moderate risk, n (%)	2616 (83.73)	1355 (69.69)	-	3971 (71.16)	
HEART high risk (%)	148 (4.73)	529 (27.20)	-	677 (12.13)	
**Research strategy**					
Cardiac catheterization (CATE), n (%)	2466 (78.93)	1783 (91.71)	490 (95.69)	4739 (84.93)	<0.001
Myocardial scintigraphy, n (%)	392 (12.54)	59 (3.02)	2 (0.38)	453 (8.12)	<0.001
Coronary angiotomography, n (%)	112 (3.58)	10 (0.50)	-	122 (2.19)	NC
Magnetic resonance imaging, n (%)	18 (0.57)	9 (0.45)	2 (0.38)	29 (0.52)	NC
Exercise test, n (%)	9 (0.28)	-	-	9 (0.16)	NC
Stress echocardiogram, n (%)	-	-	-	-	NC
**Treatment performed**					
Clinical, n (%)	1692 (54.15)	792 (40.73)	109 (21.28)	2593 (46.47)	<0.001
Angioplasty, n (%)	1068 (34.18)	912 (46.90)	377 (73.62)	2357 (42.24)	<0.001
Coronary artery bypass grafting, n (%)	364 (11.64)	240 (12.34)	26 (5.07)	630 (11.29)	<0.001

NC: not calculated due to the small number of patients; ACS: Acute coronary syndrome. NSTEMI: non-ST-segment elevation myocardial infarction; STEMI: ST-segment elevation myocardial infarction.

Regarding the evolution for the total number of patients and by group (Table 3), we have the following findings: 3% of patients presented major bleeding, being slightly less prevalent in the UA group, the same pattern followed by stroke (p-value 0.037 and 0.007, respectively).

**Table 3:** – Evolution

Variable	Unstable Angina	NSTEMI	STEMI	ACS	p-value
Evolution during the hospital stay					
Major bleeding (TIMI), n (%)	80 (2.56)	74 (3.80)	18 (3.51)	172 (3.08)	0.037
Stroke, n (%)	13 (0.41)	22 (1.13)	6 (1.17)	41 (0.73)	0.007
Infarction/reinfarction, n (%)	50 (1.60)	37 (1.90)	15 (2.92)	102 (1.83)	0.109
Death, n (%)	20 (0.64)	56 (2.88)	29 (5.66)	105 (1.88)	<0.001
**Evolution within 30 days after discharge**					
Major bleeding (TIMI), n (%)	118 (3.77)	45 (2.31)	10 (1.95)	173 (3.10)	0.004
Stroke, n (%)	195 (6.24)	87 (4.47)	19 (3.71)	310 (5.55)	0.005
Infarction/reinfarction, n (%)	254 (8.13)	160 (8.23)	24 (4.68)	439 (7.86)	0.020
Death, n (%)	35 (1.12)	78 (4.01)	28 (5.47)	141 (2.53)	<0.001
Average length of hospital stay, days	6.42	7.55	7.55	7.17	

ACS: Acute coronary syndrome. NSTEMI: non-ST-segment elevation myocardial infarction; STEMI: ST-segment elevation myocardial infarction.

Infarction/reinfarction was present in 1.8% of patients, being more prevalent in the STEMI group (p-value = 0.019). 1.9% of patients died, being more present in the STEMI group, followed by the NSTEMI group (p-value <0.001) (Figure 1).

After 30 days of discharge, 3% of patients presented major bleeding, being slightly less prevalent in the UA group (p-value 0.004). Stroke was present in 5% of patients, being somewhat less commonplace in the UA group (p-value = 0.005).

Reinfarction occurred in 8% of patients and was less prevalent in the STEMI group (p-value = 0.020). 2.5% of patients died, and it was more common in the AMI groups (p-value <0.001). The central figure summarizes the main findings described above.

## Discussion

This is the largest registry on ACS published to date. The influence of predisposing risk factors on the incidence of ACS can be seen in a detailed and consistent manner.

The main comorbidities found (hypertension, obesity or overweight, diabetes, and dyslipidemia) are present in at least half of the population and follow the global trend, with globally validated guidelines even existing for the prevention of such risk factors.^12^ Regarding obesity, the prevalence of 70% of those evaluated was observed, well above the 39% described by the WHO in 2016, which raises the level of cardiovascular risk (CVR) in our population.^13^ In 2019, a global registry already warned of obesity as a contributor to five million deaths globally.^14^Unsurprisingly, hypertension, CAD, and heart failure increase with obesity.

The presence of more than 50% of diabetic individuals is also alarming. In 2017, the Pure study warned about the correlation of dietary intake, especially high in carbohydrates, with CVD and death.^15^ Data in the literature reinforce that the increase in CVR is already present in pre-diabetics and that CVD is the main cause of death in diabetic individuals.^16^ In a cohort study conducted in the United States, diabetes accounts for an 18% increase in cardiovascular death.^17^ Our study reinforces the need for strategies for primary and secondary prevention of DM, given the prevalence of this disease in patients with CAD.

Seventy percent of the patients represented in the study were hypertensive. A meta-analysis revealed the association of hypertension with death from CAD at all ages, especially when associated with the duration of the disease and an increase of 20 mmHg in systolic pressure and 10 mmHg in diastolic pressure.^18^ Data from 2019 show that hypertension was the main cause of 1.16 million deaths, in addition to 21.5 million loss of age-adjusted life expectancy.^19^ From the perspective of such data, this record brings an alert regarding the need for measures to prevent hypertension, provide early diagnosis, and avoid therapeutic inertia.

Approximately half of the population studied (40%) had experienced at least one episode of ACS. A US cohort evaluated approximately 240,000 patients with a first ACS event and the risk of recurrence and revealed that 40% of the population was at risk of a new event within the first year of hospital discharge.^20^ A Brazilian registry of fifteen thousand patients evaluated the main factors associated with the recurrence of ACS, highlighting medication adherence as one of the main contributing factors to the recurrence of ACS. For example, 16% of those evaluated did not take any of the recommended medications, and 11.8% used only 1 of the medicines in just 4 years after the event.^21^ Therefore, we can consider poor medication adherence as a new risk factor to consider in patients.

All of the factors mentioned above can be prevented by improving public policies aimed at the primary prevention of CVD. These policies include educational campaigns on the importance of maintaining a physical exercise routine and a balanced diet. The WHO recommends practicing 150 to 300 minutes of moderate-intensity activity per week or 75 to 150 minutes of intense activity per week.^22^

Another fundamental point is proper adherence to medication, cessation of smoking, and implementation of personalized care. All of these measures should be initiated in Family Health Units, which play a crucial role in the early detection of the main risk factors for diseases in the population assisted, in addition to establishing a follow-up in the changes in the individuals’ lifestyle. It is worth noting that the isolated use of risk scores (GRACE, TIMI, and HEART) alone did not show good accuracy in distinguishing between UA and Infarction. Studies have already suggested combined classifications to increase the accuracy of angiographic diagnosis, including with the other scores presented in the registry.^23^

The performance of risk scores has been questioned. Recent evidence has shown that clinical judgment combined with high-sensitivity troponin and electrocardiogram has greater sensitivity and NPV than the TIMI score.^24^

Our clinical practice shows a high frequency of cardiac catheterization, directly reflecting the epidemiological reality of the population we serve. We observed a significantly high prevalence of pre-existing CAD among patients, approaching 60%. This high rate of previous CAD in the population justifies the frequent recurrence of catheterization as a primary diagnostic and therapeutic tool in our medical practice.

During the 30-day follow-up, the presence of major bleeding, ischemic stroke, infarction, and death associated with UA is highlighted. It is known that the diagnosis of UA is currently questioned in the literature,^25^ which expands the identification parameters but at the same time makes accurate diagnosis and immediate therapeutic management more difficult, making the patient more prone to thromboembolic events.

### Study limitations

Data collection began in 2019 when the institution did not yet use the high-sensitivity troponin kit, which was only standardized in 2021. This factor can underestimate the diagnosis of infarction, as well as overestimate the diagnosis of UA. For the composition of a large part of the record, the information was collected directly from the patients, with limitations such as the subjectivity of understanding the questioning, as well as masking of the pathological condition of the patients, since many patients who were admitted to the institution had a weak history of medical follow-up, and were even unaware of having comorbidities identified in primary care, such as hypertension and diabetes. Thus, it is estimated that the prevalence of the comorbidities mentioned above is even higher than that described in the present study. In addition, there was difficulty in classifying the interviewees regarding the practice of physical activity since the answers diverged as they were questioned again during the follow-up. Due to the possibility of unreliable representation of the data, the authors chose not to disclose this information but emphasized the importance of combating a sedentary lifestyle as a measure to prevent cardiovascular risk. It was not possible to initially classify the origin of all patients admitted to the emergency room of the service and the total duration of symptoms since the first visit in case of referral. This is due to the unavailability of SUS records with the complete evaluation of the patient until they were admitted to the emergency room of the institution. Finally, it is emphasized that the follow-up time may have been insufficient to conclude the main cardiovascular outcomes, although the results found are endorsed by the main studies on coronary syndrome in the scientific community.

## Conclusion

The above record contributes to elucidating which strategies should be used in the primary prevention of ACS, assisting in the generation of public policies for such. It is also worth highlighting the need for new studies that include other regions of the country, in order to reflect the national health reality more accurately.
